# Novel Insights in Systemic Lupus Erythematosus and Atherosclerosis

**DOI:** 10.3389/fmed.2017.00262

**Published:** 2018-01-29

**Authors:** Vítor Teixeira, Lai-Shan Tam

**Affiliations:** ^1^Rheumatology Department, Centro Hospitalar de Lisboa Norte, EPE, Hospital de Santa Maria, Lisbon, Portugal; ^2^Department of Medicine and Therapeutics, The Prince of Wales Hospital, The Chinese University of Hong Kong, Sha Tin, Hong Kong

**Keywords:** systemic lupus erythematosus, atherosclerosis, cardiovascular events, physiopathology, biomarkers

## Abstract

**Introduction:**

The systemic inflammatory nature of systemic lupus erythematosus (SLE) is well patent not only in the diverse clinical manifestations of the disease but also in the increased risk of premature atherosclerosis and cardiovascular events (CVE), making SLE one of the most complex diseases to study and manage in clinical practice.

**Aim:**

To travel from old aspects to modern insights on the physiopathology, new molecular biomarkers, imaging methods of atherosclerosis assessment, and the potential treatments of atherosclerosis in SLE.

**Methods:**

We conducted a literature search using PubMed database and performed a critical review.

**Conclusion/discussion:**

Several developments have taken place in the understanding of the relationship between SLE and premature atherosclerosis. Nevertheless, cardiovascular diseases are still the major cause of reduced life expectancy in SLE and the main cause of death. The lack of standardization methods for the imaging assessment of atherosclerosis in SLE and the multifactorial nature of the disease are well patriated in the difficulty of achieving consistent and reproducible results among studies that focus in cardiovascular risk assessment and prediction. A raising number of molecular biomarkers of atherosclerosis have been proposed, but the combination of several biomarkers and risk factors may better estimate cardiovascular disease risk. Moreover, the development of effective therapies to prevent progression of atherosclerosis and CVE shall address systemic inflammation.

## Introduction

Atherosclerotic lesions were initially thought to result mainly from an abnormal accumulation of lipoproteins associated with a disorganization of the intima and deformation of the arterial wall (1). However, since inflammation was implicated in the pathogenesis of atherosclerosis, several studies have focused on the immunologic aspects of atherosclerosis. Alterations of specific immune functions play a pivotal role in all stages of atherosclerotic plaque development from its initiation to progression (2). Diseases characterized by systemic inflammation, like systemic lupus erythematosus (SLE), have been strongly linked to accelerated atherosclerosis and increased cardiovascular morbidity and mortality. This association is only partially explained by the presence of traditional Framingham cardiovascular risk factors (3).

About half a century ago, the reported mortality of patients with SLE was 50% in the first 5 years after diagnosis and was mainly related to disease activity (4). In Europe, the 5-year survival of SLE is currently at 95% (5) and the 10-year survival is around 90% (6). Despite this improvement of survival in the first years of disease, the pattern of mortality of SLE is for a long time known to be bimodal, with a late peak of mortality largely due to cardiovascular disease (7). This peak has remained almost unchanged in recent years, contributing to an important reduction in the average life expectancy of 20 years in SLE (8). In fact, community-based studies have found that cardiovascular diseases are currently the main cause of death in SLE (9).

## Review Methodology

We conducted a selected sampling of the literature, using PubMed as a database, and we performed a critical review on the relationship of lupus and atherosclerosis. We focused on cardiovascular events (CVE), atherosclerosis physiopathology, molecular and imaging biomarkers, and treatment strategies for atherosclerosis prevention in SLE.

## Results

### Cardiovascular Events

Patients with SLE have several comorbidities that are related not only to the disease itself but also to treatments and associated autoimmune diseases, such as antiphospholipid syndrome (APS), making it difficult to stablish the exact etiology of CVE in many patients. Nevertheless, data suggests that SLE is *per se* a strong independent risk factor for the development of CVE, comparable even to type I diabetes mellitus (DM) (10). A large study that included 1874 SLE patients estimated a risk of 2.7-fold in acute CVE (stroke, myocardial infarction, angina, coronary intervention, and peripheral vascular disease) relative to the risk that would be expected based on the Framingham risk score (11). This risk was remarkably higher in the sub-group of younger women aged 35–44 years, in whom it reached a 50-fold risk (12). In the Toronto lupus cohort, the mean age of myocardial infarction was 49 years compared with the peak years of the general population of 65–74 years (13). Coronary artery disease is responsible for 30% of deaths in SLE (14). Also of concern, SLE patients have striking poorer outcomes after percutaneous coronary intervention (PCI) than non-SLE patients, being more likely to suffer a new myocardial infarction (15.6 versus 4.8%, *p* = 0.01) and repeat PCI at 1 year after the first PCI (31.3 versus 11.8%, *p* = 0.009) (15). Moreover, SLE patients have lower 3-year survival and higher need of re-intervention after coronary artery bypass grafting (16). A recent report from the USA Nationwide Inpatient Sample highlighted that from 1996 to 2012 there was an increase in the rates of hospitalization due to acute myocardial infarction and ischemic stroke in SLE patients, despite a reduction in hospitalization due to unstable angina (17).

The relative risk of cerebrovascular events in SLE is lower than for coronary events, but twice higher the risk in the general population. It is also particularly higher in the first year after diagnosis and in women younger than 50 years (18). Despite that and after exclusion of APS, the absolute risk is considered low before the age of 60, and the highest rate is found in those individuals ≥60 years old (18). SLE patients without clinical neurological involvement also have more visible perivascular spaces and white-matter hyperintensities on magnetic resonance imaging (MRI) than controls, suggesting a subclinical compromise of the cerebral small vessel integrity (19).

The risk of peripheral artery occlusive disease (PAOD) is also high and has been reported to be ninefold higher than the general population, and higher in the first year after diagnosis; afterward it tends to decline (20). As in myocardial infarction and ischemic stroke, the relative risk was particularly higher in younger women, principally those less than 34 years (HR = 47.6, 95% CI = 26.8–84.4, *p* < 0.001) (20). In a Spanish study, age was found to be the only independent variable of increased PAOD risk among the traditional risk factors (21). Apart from age, Hassan and colleagues, additionally found a positive correlation with DM, dyslipidaemia, smoking, extended duration of steroid use, the Systemic Lupus International Collaborating Clinics Damage Index, use of azathioprine or warfarin, and plasma levels of thrombotic variables (22). No association was found with age in a study by Bhatt and colleagues, while dyslipidaemia was the only traditional risk factor in this study associated with increased risk of PAOD (23).

### Mechanisms of Atherosclerosis

**Figure 1:**
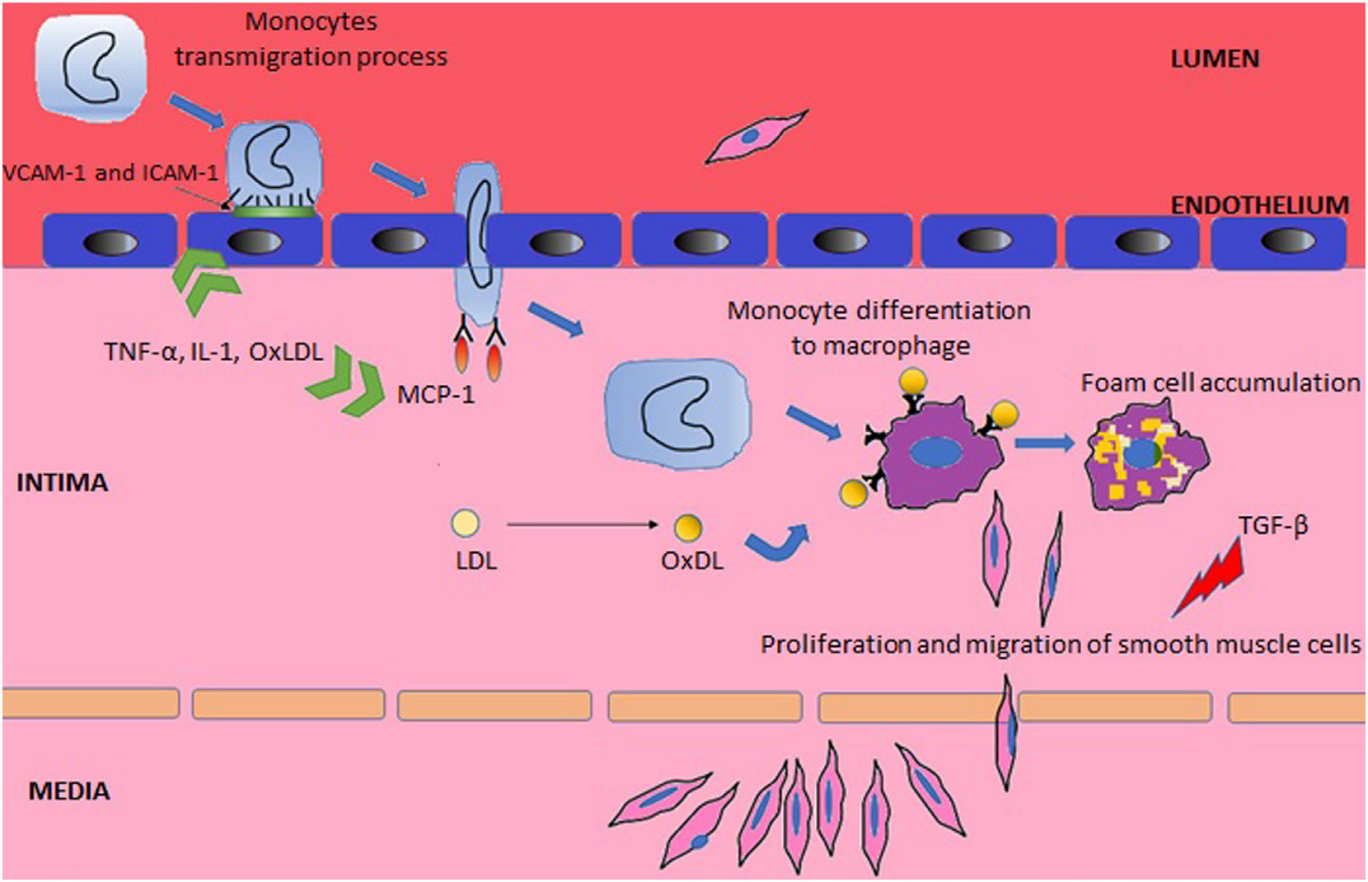
Main steps in the pathogenesis of atherosclerosis.

Systemic lupus erythematosus may affect the integrity and repair mechanisms of endothelial cells through direct binding of antibodies to endothelial cells or deposition of circulating immune complexes (24). The consequent endothelial damage promotes atherogenesis, which has been divided into three stages (Figure 1) (2). The inflammatory process triggers the first stage, which consists of expression of surface molecules in the vascular wall that are responsible for adhesion of leukocytes [such as vascular cell adhesion molecule-1 (VCAM-1) and intercellular adhesion molecule-1 (ICAM-1)], their rolling (selectins) and attachment (integrins) (2, 25). In the second stage, the adherent leukocytes migrate across the intima layer and penetrate the media layer (2). The transmigration process is mediated by monocyte chemotactic protein-1 (MCP-1). In mice models of accelerated atherosclerosis, the knockout of MCP-1 is associated with a reduction of atherosclerosis (26), while in humans, elevated circulating levels of MCP-1 correlate to increased carotid intima-media thickness (IMT) (27). l-Homocysteine is another promoter of the leukocyte recruitment by inducing expression of MCP-1 and interleukin-8 (28). In SLE, the elevation of plasma homocysteine concentrations after oral methionine load is associated with elevated markers of endothelial damage and platelet activation, inducing a prothrombotic tendency (29).

Tumor necrosis factor (TNF)-α, interleukin (IL)-1, and oxidized low-density lipoprotein (OxLDL) have an important role in the two first stages of atherosclerosis, as they upregulate adhesion molecules and MCP-1 (26, 30). Conversely, the transforming growth factor (TGF) supresses smooth muscle and endothelial cell proliferation (31). Decreased levels of TGF-β was associated with premature atherosclerosis in lupus-prone mice (32), supporting the hypothesis that downregulation of this cytokine can lead to arterial wall dysfunction with subsequent atherosclerosis development.

Finally, in the last stage, there is formation of macrophages foam cells and replication of smooth muscle cells leading to plaque propagation and rupture (2). This complex stage starts with the entrapment of low-density lipoprotein (LDL) in the subendothelial space where it is exposed to reactive oxygen species and then converted to OxLDL (33). The exposure of endothelial cells to OxLDL results in production of more MCP-1 and macrophage colony-stimulating factor, which further contribute to monocyte binding, chemotaxis, and differentiation to macrophages (34). In addition, when OxLDL is being exposed to macrophages, it contributes toward the inhibition of the phagocytosis of apoptotic cells and promotes the expression of the scavenger receptor CD36, which further increases the phagocytosis of OxLDL (35), leading to foam cells formation (36).

Oxidized low-density lipoprotein, unlike native LDL, can also form complexes with β2-GPI, which are significantly elevated in SLE with or without APS (37). The presence of IgG β2-GP1 increases OxLDL uptake by macrophages (38). Both, elevated levels of circulating OxLDL and of antibodies against OxLDL, are more common in SLE patients with CVD, than patients without CVD (39). Elevation of antibodies against epitopes of OxLDL has also been associated with maximum IMT and progression of atherosclerosis (40). Nevertheless, the heterogeneity among Ig subclasses and epitopes specificity and affinity might explain diverse properties for oxLDL antibodies (41). In fact, only IgG antibodies are thought to promote atherogenesis, while IgM antibodies seem to protect atherosclerosis prone mice from inflammatory oxidized moieties (42).

Tumor necrosis factor-α also plays a role in the last stage as it suppresses lipoprotein lipase synthesis and thus inhibits the metabolism of triglycerides and very LDL (43). The protective mechanism of efflux of cellular cholesterol is thought to be impaired in SLE, as the responsible enzyme for this process, cholesterol 27-hydroxylase, has been showed to be decreased in incubated human monocytes and aortic endothelial cells of SLE patients (44).

Notably, one of the most important overexpressed cytokine in SLE, interferon (IFN)-γ, upregulates several pro-atherogenic processes such as the production of lipid mediators, platelet-activating factors and eicosanoids, antigen presentation, and synthesis of TNF-α and IL-1 (45). The activation of toll-like receptors 7 and 9 is responsible for the upregulation of IFN-α expression (46). Mice models exposed to IFN-α have increased apoptosis, dysfunctional endothelial progenitor cells (EPCs), and reduced number of endothelial and smooth muscle cells (47). The pro-atherogenic effect of IFN is substantiated in other mice experiments, where a correlation was found between the depletion and dysfunction of EPCs and excessive type I IFN levels (48). The levels of these EPCs correlate inversely with cardiovascular risk in the general population (49). Furthermore, IFN is also responsible for plaque instability, as it inhibits the growth of smooth muscle cells, endothelial cells, and the production of collagen trough mechanisms that are not fully understood (50).

Accelerated atherosclerosis in SLE may be also related with the presence of antiphospholipid antibodies (aPL), known to increase the risk of thrombosis in SLE through several mechanisms. APL interact with endothelial cells and monocytes inducing a pro-inflammatory and pro-coagulant phenotype (51, 52) and activate the complement, that generates C5a which then activates neutrophils and expression of tissue factor (responsible for the initiating of the extrinsic coagulation cascade) (53). Specific autoantibodies against phospholipids also induce the expression of adhesion molecules, such as ICAM-1, VCAM-1, and E-selectin (54). Other mechanisms of atherogenesis include augmentation of the production of intracellular ROS (55) and promotion of lipid peroxidation of lipoproteins by reducing the activity of paraxonase 1 activity (56).

A new input for the understanding of the relationship between lupus and atherosclerosis came with the identification of NETosis as a key pathophysiological element. NETosis is a type of cell death pathway that results from externalization of chromatin fibers decorated with granule-derived antimibrobial peptides and is one of the defense mechanisms of neutrophils against pathogens (57). Neutrophil extracellular traps (NETs) contain many proinflammatory antimicrobial molecules, such as neutrophil elastase, IL17, human cathelicidin (LL 37), myeloperoxidase, histones, and MMP-9 (58–60). Many of these molecules induce endothelial cell death and vascular disfunction.

In SLE, there is a deficient clearing of these NETs, which increases production and release of type I IFN and further enhances NETosis (59). Impaired degradation of NETs is presumably due to the presence of antibodies against DNase (deoxyribonuclease) I, the main degrader of NETs, or due to the presence of anti-NET autoantibodies that protect NETs from degradation (61). NETs also lead to increased inflammasome activation, increasing the synthesis of activated IL1β and IL-18, which induce a positive loop of NET formation (62).

Low-density granulocytes (LDGs), a subtype of neutrophils prevalent in SLE, are particular prone to predispose NETosis (63). LDG NETs are deleterious for the endothelium and thus thought to contribute for accelerated atherosclerosis in SLE, as they synthesize increased levels of pro-inflammatory cytokines, mainly IFN-α and disrupt the differentiation of EPC to mature endothelial cells (59, 60, 63). LDGs also display significant increases in mitochondrial ROS production (63). The oxidation of mitochondrial DNA (mtDNA), mediated by ROS, allows migration of mtDNA to the cellular surface, thereby triggering potent pro-inflammatory and interferogenic responses (63). In fact, mitochondrial ROS are required for maximal NET stimulation in animal models. There is additional evidence favoring a role of NETosis in atherosclerosis progression, coming from studies that demonstrated the presence of NETs in atherosclerotic plaques (64) and that the inhibition of NET formation protected mice from atherosclerosis and mitigated type I IFN response (65).

### Molecular Biomarkers

The progressive knowledge on the physiopathology of atherosclerosis has led to the search for potential biomarkers of cardiovascular disease and atherosclerosis progression, derived mainly from plasma measurements. In general, most molecular biomarkers studied so far have low to modest prediction value of atherosclerosis progression and CVE (66). Moreover, strong epidemiological studies that make direct comparisons of the value of different biomarkers in patients with SLE are lacking. Examining the individual role of each biomarker in the pathophysiology of atherosclerosis in SLE and the predictive value for atherosclerosis and CVE is not the objective of this review, and some of them have been previously discussed in Section “Mechanisms of Atherosclerosis.” Table 1 resumes some of the available literature regarding several molecular biomarkers and associations found with CVE and mortality and correlation with imaging markers of atherosclerosis in patients with SLE. We will focus on three newly identified atherosclerosis biomarkers in SLE: pentraxin-3 (PTX3), pro-inflammatory HDL (piHDL), and endocan. PTX3 is produced from mononuclear phagocytes, myeloid-derived dendritic cells, and endothelium cells in response to local inflammation, being considered a biomarker of local vascular inflammation (67). Levels of PTX3 are increased in SLE and correlate with disease activity (68). Furthermore, levels of PTX3 correlate with other indicators of endothelial dysfunction such as the soluble VCAM-1 and vWf (69). These data suggest that PTX3 could be a novel biomarker for premature atherosclerosis in SLE.

**Table 1:** Molecular biomarkers and associations with cardiovascular events and mortality and atherosclerosis imaging surrogates.

Molecular biomarkers	Associated with cardiovascular events	Associated with cardiovascular mortality	Correlation with imaging markers of atherosclerosis (presence or progression)	Reference
Absence of thrombocytopenia	+			(70)
Adiponectin			±	(71, 72)
Annexin A5			+	(73)
Anti-apoA-I			+	(74)
Asymetric dimethtlarginine	+		+	(75, 76)
C3 complement			+	(77, 78)
CRP	+	+	±	(70, 79)
Endocan			+	(80)
EPC			−	(81)
Erythrocyte nitric oxide			−	(82)
E-selectin			+	(79)
Fatty-acid-binding protein 4			+	(83)
Homocysteinemia	+		+	(84, 85)
				(86–89)
ICAM-1			+	(79)
IgM anti-malondialdehyde			−	(90)
IgM anti-phosphorylcholine			−	(90)
IL-6	+			(79)
Leptin			±	(72, 79)
P-C4d	+	+		(91)
piHDL			+	(92)
PON-1 activity	−			(93)
sCD40L	+			(94)
TGF-β			−	(32)
TWEAK	+		+	(66, 79)
Type I IFN	+			(66, 79)
VCAM			+	(79)
VEGF	+		+	(79, 95)
Vitamin D			−	(96–98)
VWf	+			(70)
Whole blood viscosity	+		+	(82, 83, 99)

*+, positive correlation/association; −, negative correlation/association; ±, ambiguous evidence among the studies referenced*.

*Anti-apoA-I, anti- apolipoprotein A1; CRP, C-reactive protein; EPC, endothelial progenitor cell; ICAM-1, intercellular adhesion molecule 1; IFN, interferon; IL, interleukin; P-C4d, Platelets bearing complement protein C4d; piHDL, pro-inflammatory high-densitity lipoprotein; PON-1, paraxonase 1; sCD40L, soluble CD40 ligand; TGF, transforming growth factor; TWEAK, tumor necrosis factor-like weak inducer of apoptosis; VCAM-1, vascular cell adhesion molecule-1; VEGF, vascular endothelial growth factor; vWf, von Willebrand factor*.

Cholesterol HDL is generally considered atheroprotective, but can lose its protective effect against the oxidation of LDL. This happens when it is converted from the usual anti-inflammatory form to the piHDL form, which usually occurs in the advent of chronic inflammatory states, like in SLE (92). The piHDL form is associated with progression of carotid plaques (odds ratio 16.1, *p* < 0.001) and IMT (odds ratio 2.5, *p* = 0.02) in patients with SLE (66, 100).

Endocan is an indicator of angiogenesis and endothelial cell activation (101) and participates in the recruitment, adhesion, and migration of leukocytes across the endothelium (102). In SLE, one study addressed its effects in atherosclerosis and concluded that serum levels were greater in these patients than in controls; in addition, endocan levels correlated positively with cIMT (*r* = 0.469, *p* < 0.01) (80).

Combinations of biomarkers may predict more accurately the atherosclerosis risk and CVE risk than isolated biomarkers (66). For SLE, it has been developed the Predictors of Risk for Elevated Flares, Damage Progression, and Increased Cardiovascular Disease in SLE (PREDICTS) model, which includes four inflammatory biomarkers (homocysteine, piHDL, TWEAK, and leptin) and two risk factors (age and diabetes) (66). In the population studied, none of the individual biomarkers had a good balance of strong positive predictive value and negative predictive value plus high specificity and sensitivity. On the contrary, the complete panel had a better predictive capacity for the longitudinal presence of plaque in SLE patients than did the individual parameters. Remarkably, a high-risk score conferred a 28-fold increased odds ratio of carotid plaque and an 8-fold increased odds ratio for cIMT progression, both with statistical significance.

Currently, however, combining several biomarkers has limited use in daily clinical practice, is probably not cost effective and needs to be validated in other SLE cohorts. Moreover, evidence-based treatment decisions according to a determinate risk score will be needed before wide use.

### Imaging Assessment of Atherosclerosis

Subclinical atherosclerosis is an early finding in SLE patients and an important predictor of cardiovascular risk and morbidity (84). Subclinical atherosclerosis should not be overlooked, because it contributes to peripheral embolism, pre-hypertension, or hypertension and increased left ventricular afterload that can result in left ventricular hypertrophy and diastolic dysfunction (103, 104).

To access subclinical atherosclerosis, several non-invasive imaging surrogate indexes have been used for evaluation of morphological or functional arterial alterations: IMT, detection of atherosclerotic plaques, flow-mediated dilation (FMD), pulse wave velocity (PWV), and myocardial perfusion studies using single photon emission computed tomography (SPECT) dual isotope or MRI (Table 2). For large-scale populations, cIMT has been the most widely used, being able to predict stroke and myocardial infarction in the general population (105, 106). The detection of atherosclerotic plaques is, however, a better predictor of cardiovascular risk than IMT alone (107, 108). For early stages of atherosclerosis, IMT may be a more sensitive marker (109).

**Table 2:** Summary of atherosclerosis imaging surrogates in SLE.

	Procedure assessment	Frequency estimates in SLE	Cardiovascular disease prognostic significance in SLE
FMD	Doppler ultrasound measurement of vasodilation in response to an acute increase in blood flow, typically induced by cuff inflation and then deflation and after administration of nitroglycerin. It is usually measured at the brachial artery level (110)	Reported to be significantly lower in patients with SLE without clinical CV disease than healthy controls in a case-control study (mean 3.72 ± SD 28% versus 4.63 ± 3.1%, *p* = 0.032) and on a meta-analysis (SMD = 0.926, 95% CI 1.414 to −0.438, *p* < 0.001) (111)	Predictive value for CVE is not clear
PWV	Velocity at which the pressure waves, generated by the systolic contraction of the heart, propagate along the arterial tree. Usually measured at the carotid-femoral or carotid-radial levels (112)	Reported to be higher in patients with SLE than healthy controls on a meta-analysis (SMD = 0.56, 95% CI 0.3–0.82, *p* = 0.834) (113)	Predictive value for CVE is not clear, but a higher PWV has been associated with other classic cardiovascular risk factors such as age, body mass index, and hypertension (113–115)
IMT	Distance between lumen-intima and media-adventitia interfaces, usually measured with high-resolution ultrasonography (116)	Elevation of carotid IMT was found in 28% of patients with SLE (40)	High carotid IMT are predictive of future CVE (HR 1.35, 95% CI 1.12–1.64, *p* < 0.01) in female patients with SLE without previous CV events (117)
Plaques evaluation	Two-dimensional measurement of plaque (cross-sectional area of plaques viewed in a longitudinal plane) or 3-dimensional measurement of plaque volume (109)	Carotid plaques are 2.4 times more frequent in SLE than the general population, and 5.6 times more prevalent in those <40 years (84)	Carotid plaque was predictive of future CVE (HR 4.26, 95% CI 1.23–14.83) in female patients with SLE without previous CV events (117)
Myocardial perfusion studies	Usually uses SPECT or PET to assess the blood flow to the myocardium when it is stress by exercise or medication (118, 119)	Myocardial perfusion defects have been detected in 40% of women with SLE (118)	Myocardial perfusion defects in SPECT predict a 13-fold increased risk of CVE (120)

*CI, confidence interval; CV, cardiovascular; CVE, cardiovascular events; FMD, flow-mediated dilation; HR, hazard ratio; IMT, intima-media thickness; PET, positron emission tomography; PWV, pulse wave velocity; SD, standard deviation; SLE, systemic lupus erythematosus; SMD, standardized mean difference; SPECT, single photon emission computed tomography*.

#### Flow-Mediated Dilatation of the Brachial Artery

Vascular stiffness is usually estimated from FMD or PWV using vascular ultrasonography. A higher pulse pressure to achieve a certain degree of vessel distension suggests a lower vessel distensibility and thus a higher stiffness (121). Impaired FMD is thought to represent an early stage in the process of atherogenesis. SLE patients have significantly lower FMD, not fully explained by classic cardiovascular risk factors (111). Lupus nephritis, higher disease activity measured with SLE Disease Activity Index (SLEDAI), and higher levels of CRP have been correlated with impaired brachial endothelial function (122). Aortic stiffness, evaluated directly with transoesophageal echocardiography, is higher in SLE patients with hypertension, but normotensive SLE patients also have higher aortic stiffness than normotensive controls (123).

#### Pulse Wave Velocity

Systemic lupus erythematosus patients also have higher PWV (carotid-femoral or carotid-radial) than controls, but the predictive value for CVE is not clear (113). Despite that, PWV velocity has been associated with other classic cardiovascular risk factors such as age, body mass index, and hypertension (113–115, 124).

#### Intima-Media Thickness

Most studies determine IMT at the carotid level due to the better accessibility, but the prevalence, characteristics, risk factors, or predictors of aortic atherosclerosis are probably similar to carotid atherosclerosis (125–127). There is considerable heterogeneity regarding studies protocols in the method of measuring IMT in terms of the carotid segment investigated, unilateral/bilateral measurements, far wall, near wall, or both walls measurements and expression and mean or a maximal IMT. Nevertheless, there is clear evidence for a significant increase of cIMT in SLE patients, as showed in a systematic review and meta-analysis (116). In women with SLE without previous CVE, the cIMT at baseline was predictive of future CVE (117). This was especially true in the presence of concomitant carotid plaques. As for aortic IMT, values are also higher in SLE patients than age- and gender-matched controls (117).

#### Imaging Evaluation of Atherosclerotic Plaques

In SLE, carotid plaques are reported to be 2.4 times higher than the general population, with a peak of 5.6 times higher among patients <40 years (84). Patients at higher risk also include those with longer disease duration, higher damage index score, and less aggressive immunosuppressive therapies (84). Likewise, coronary artery calcifications evaluated through computed tomography appear to be more pronounced in younger SLE patients when compared to age-matched controls, but also in patients with increased disease activity (128). When assessing the risk of CVE related to atherosclerotic plaques, it has been suggested that quantification of plaques may reflect better the extent of atherosclerosis, rather than only assessing the presence or absence of plaques (109).

#### Myocardial Perfusion Studies

Using SPECT, myocardial perfusion defects were detected in 40% of women with SLE (118). A 13-fold increased risk of CVE was found in patients with SLE who had perfusion abnormalities on SPECT after a mean follow-up of 8.7 years (120). In other study using positron emission tomography, myocardial blood flow was measured in the resting phase and after administration of adenosine (hyperemic phase) (119). Inclusion criteria were patients suffering from either SLE or rheumatoid arthritis, with normal or minimally diseased coronary arteries (<20% luminal diameter reduction) at angiography and without cardiovascular risk factors. Overall, the study found that myocardial blood flow during adenosine hyperemia and coronary flow reserve were significantly blunted in patients compared with controls. Despite the small sample size, the study alerted for the fact that a reduced coronary flow reserve can be found in the absence of significant coronary artery disease and might be the result of prolonged inflammation that precedes premature coronary artery disease.

Myocardial perfusion studies using MRI have, additionally, led to conclude that SLE patients tend to have a distinct and more diffuse pattern of coronary artery wall contrast enhancement than coronary artery patients without SLE or healthy controls. These differences were explained by a more diffuse vascular inflammation in SLE (129).

#### Future Perspectives

Carotid ultrasound will remain probably the most used imaging method to assess atherosclerosis as it is relatively non-inexpensive and non-invasive and already validated. Still, there is great variation in IMT acquisition and analysis among studies. Although for SLE no specific recommendations exist for assessment of IMT, compliance with other published guidelines, such as from the American Society of Echocardiography cIMT task force (2012) or the Advisory Board for the “watching the risk symposia” at the European Stroke Conferences (2011), can help to standardize methods of assessment, improve the quality of future studies, and facilitate the comparison of results among them (130, 131). Similarly, the International Brachial Artery Reactivity Task Force guidelines should be applied when assessing FMD of the brachial artery (132). A combined approach, for example, assessment of IMT and plaque, may improve sensibility and risk prediction.

### Predictive Factors of Premature Atherosclerosis

#### Traditional Risk Factors

As described elsewhere in this article, traditional risk factors could not account for the total burden of CVE in SLE; however, they are still very important and frequent in this population. In the Hopkins Lupus Cohort, several traditional risk factors like age, hypertension, obesity, and cholesterol >200 mg/day were showed to be the main predictors of coronary artery disease (109). Despite being a relatively young SLE cohort (average 38.3 ± 12.1 years), half the patients had three or more coronary artery disease risk factors, largely exceeding that of other same-nationality populations matched for age, race, and sex. Other cohorts have also shown, compared to healthy controls, a higher prevalence of sedentary lifestyle and obesity (85), older age (12, 14, 133), hypertension (14), dyslipidemia (12), and DM (85).

Tobacco use has also been linked to coronary events in SLE (7, 134), although other studies failed to prove this (135). Differences among the cardiovascular risk associated with smoking have been hypothesized to be due to race specific effects, as one study showed that history of smoking in SLE was associated with higher mean IMT measures in black women, but not white woman (136).

Disability caused by SLE can limit the ability to exercise. A sedentary life style has been identified as one of the most common risk factors for coronary artery disease, being present in 70% of patients (135). This was attributed to disease and treatment-related variables such as fatigue, anemia, arthritis, and avascular necrosis of bone.

#### Lupus Associated Risk Factors

##### Autoantibodies

Patients with primary aPL have not only an increased thrombophilic profile but also thicker cIMT than controls (137). In SLE, experimental models of atherosclerosis also support the role of aPL in the development of atherosclerotic plaques and a correlation with cIMT or carotid plaques has been demonstrated (138–140). Nonetheless, these associations are not reported in other large cohorts (141–143) and some studies even found a protective role for aPL in atherosclerosis in LDL receptor-deficient mice (144). These conflicting evidence warrants further studies to clarify the influence of aPL in atherosclerosis.

Antibodies against OxLDL, although not usually determined in clinical practice, have also been correlated with lupus activity and cIMT (145).

Whether anti-double-stranded DNA (anti-dsDNA) antibodies may contribute to premature atherosclerosis remained controversial as there are studies reporting positive association (140, 146), while other studies cannot find any association between cIMT or presence of carotid plaques (133, 147). The levels of complement were not found to correlate with cIMT (147).

##### Disease Activity and Clinical Manifestations

Differences among studies regarding disease activity indices are remarkable. In a Japanese SLE population, the SLEDAI was associated with cIMT in a dose-dependent manner (148). One Italian study found that active disease at baseline according to the European Consensus Lupus Activity Measurement index was predictive of carotid plaque and thickened mean IMT measured after 5 years of follow-up (40). Another Italian study did not show a correlation of cIMT with the SELENA-SLEDAI index measured in the preceding 3 months after cIMT assessment (147). In a study by Manzi and colleagues, the use of the Systemic Lupus Activity Measure (SLAM) index at the time of ultrasound examination was even inversely related to the presence of atherosclerotic plaque (109). These variances can be due to differences among the used activity indices and the time of assessment of disease activity and ultrasound. It has been proposed that studies should consider average activity scores over a long period of time, rather than single measures, as they may reflect better lupus activity and its relationship to atherosclerosis (20).

Neurolupus was found to be a strong predictor of CVE, with a 2.21 hazard ratio for psychosis (95% CI 1.10–4.44, *p* < 0.001) and 1.85 for seizures (95% CI 1.00–3.24, *p* = 0.007), suggesting it could also be a risk factor for accelerated atherosclerosis (149).

As for lupus nephritis, one study showed that cIMT, unlike FMD, wasn’t significantly different between SLE patients with and without nephritis (122). In a different study, women with nephrotic syndrome followed prospectively for 3 years were more likely to have progression of atherosclerosis, defined as increase in the cIMT >0.15 mm and/or an increase of the plaque score (relative risk = 4.22, 95% CI = 2.18–8.15, *p* = 0.22) (150).

### Therapeutic Strategies

Large-scale randomized trials focusing on the prevention of atherosclerosis and CVE in SLE are lacking. Additionally, there are no current guidelines for the treatment and prevention of atherosclerosis in SLE. The available studies have mainly focused on the use of immunosuppressants and treatments for traditional cardiovascular risk factors. The results from studies of the effect of several drugs on atherosclerosis and CVE in SLE are summarized on Table 3.

**Table 3:** Summary of the effects of several drugs on atherosclerosis and CVE in SLE.

Drug	Effect	
	Animal studies	Clinical studies
Corticosteroids		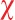 Promote cardiovascular risk factors, such as hypertension, hyperglycemia, dyslipidaemia, and obesity (151) 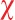 Have been associated with higher prevalence of atherosclerotic plaques and thickened cIMT (10, 109, 148, 152) 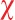 Increase the risk of CVE (11).

Statins	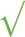 Decrease several molecules associated with atherosclerosis: IL-6, TNF-α, IFN-γ, IL-8, P-selectin (153, 154)	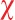 Treatment with statins did not show a significant effect in cIMT and plaque development (155, 156) 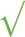 Decrease of plasma levels of MCP-1, high-sensitivity CRP, and trombomodulin (157, 158) 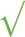 Atorvastatin possibly reduces carotid-femoral PWV (159)

ACEIs/ARBs		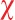 The cumulative occurrence of CVE was not shown to be statistically significant in lupus nephritis patients treated with ACEIs/ARBs (160) 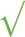 ACEI non-use has been associated with carotid plaque area (161)

Aspirin		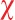 No effects on atherosclerosis biomarkers, such as homocysteine, high-sensitivity CRP, soluble vascular cell adhesion molecule 1, P-selectin, and thrombomodulin (158) 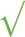 The association with HCQ has synergistic thromboprotective effect (162, 163) 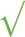 Reduces CVE in aPL positive patients (164)

HCQ	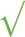 Prevents the development of endothelial dysfunction *via* reduction of reactive oxygen species (165) 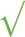 Inhibits platelet aggregation and activation mediated by aPL (168)	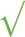 Favorable effects on lipid and glycemic control (166, 167) 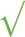 Reduces the risk of thrombovascular events (162, 163, 169, 170) 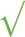 Is associated with lower progression of carotid plaque and aortic stiffness (170)

MMF	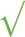 Reduces pro-inflammatory and metalloproteinase genes expression (171) 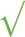 Inhibits CD4+ T-cell activation and infiltration to atherosclerotic lesions (173)	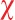 No clear effect on progression of cIMT or coronary calcification (172)

Azathioprine		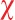 Linked to higher risk of CVE (40, 138, 174, 175)

Cyclophosphamide		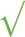 Has been associated with a lower prevalence of abnormal aortic IMT and plaques (123)

Cyclosporine A		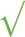 Possibly protective against increased cIMT (148)

Antibodies against BAFFR	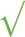 Reduce atherosclerosis in mice (176) 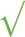 Depletes B2 cells subtype and preserves B1 cells subtype (176–178) 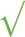 Prevents thrombosis in antiphospholipid syndrome (179)	

CD20-specific monoclonal antibodies	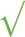 Significantly decreased atherosclerosis in mice (180) 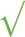 Reduce the IgG type anti-OxLDL antibodies and the accumulation of B-cells, macrophage, and T-lymphocytes in atherosclerotic plaques (180)	

Vitamin D	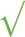 Decreases the production of pro-inflammatory chemokines and the quantity of inflammatory effector cells in atherosclerotic plaques (181) 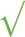 Vitamin D deficiency hampers vascular repair and reduces endothelial disfunction (186) 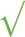 Vitamin D deficiency increases expression of type I IFN (186)	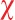 No protective effect of supplementation in atherosclerosis has been showed in SLE, despite that vitamin D deficiency has been linked to premature atherosclerosis (96–98, 182–185)

*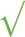 Beneficial effects; 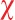 detrimental/no effects*.

*ACEIs, angiotensin-converting enzyme inhibitors, aPL, antiphospholipid antibodies; ARBs, angiotensin II receptor antagonist; BAFFR, B-cell activating factor receptor; cIMT, carotid intima-media thickness; CRP, C-reactive protein; CVE, cardiovascular events; HCQ, hydroxychloroquine; IFN, interferon; IL, interleukin; IMT, intima-media thickness; MCP-1, monocyte chemotactic protein-1; MMF, mycophenolate mofetil; OxLDL, oxidized low-density lipoprotein PWV, pulse wave velocity; SLE, systemic lupus erythematosus; TNF-α, tumor necrosis factor α*.

#### Corticosteroids

There has been a long debate on the theoretical dual action of corticosteroids in atherosclerosis. Since inflammation plays a significant role in atherogenesis, one might think that the anti-inflammatory action of corticosteroids would reduce this risk. On the other hand, corticosteroids enhance classical cardiovascular risk factors such as hypertension, hyperglycemia, dyslipidaemia, and obesity (151). Different variables such as duration of treatment, cumulative, average (all time, only few years) or maximum doses have been evaluated in different studies, which can in part explain the mixed results. A positive correlation has been found with both duration of treatment (152) and cumulative dose (10, 148, 187).

When addressing the causative role of corticosteroids, one shall also consider that cumulative disease activity and some clinical phenotypes, such as nephritis and neuropsychiatric lupus, often warrant high doses of corticosteroids and have also been associated with more atherosclerosis in SLE and thus could be confounding factors. After adjusting for all these confounding factors, Manzi and colleagues demonstrated that a longer treatment duration with prednisolone (but not current use, maximum dose, or cumulative dose) was an independent determinant of carotid plaque, while disease activity (SLAM) determined at the time of the detection of the plaque was found to be inversely related to the presence of plaque (109). Completely different results were obtained by another study that reported current disease activity (SELENA-SLEDAI) and current dose of corticosteroids (>20 mg/day) were associated with a higher cardiovascular risk (11). Finally, it has been purposed that the action of corticosteroids may be dose-dependent, as one study in pediatric SLE showed an association between the highest and lowest cumulative doses of corticosteroids and a higher cIMT, while moderate cumulative doses were associated with a decreased cIMT (188).

#### Statins

Statins theoretical benefits are related to their effect in decreasing proinflammatory cytokines and chemokines such as IL-6, IL-8, TNF-α, and MCP-1 (153, 154, 157, 189). In a Chinese randomized, double-blind, placebo-controlled trial, low-dose rosuvastatin (10 mg/day) was associated with reduction of LDL, CRP, P-selectin, and thrombomodulin (158). Moreover, atorvastatin (20 mg/day) therapy for 8 weeks has shown to reduce arterial stiffness of SLE female patients who had baseline pathological carotid-femoral PWV, although this was only significant for middle-aged patients (36–59 years) (159). Atorvastatin at a dose of 40 mg/day may stabilize the coronary artery calcium score, although it did not ameliorate perfusion defects in myocardium SPECT (190). Results from the 2-year double-blinded Lupus Atherosclerosis Prevention Study were, unfortunately, more disappointing (155). In this study, 200 SLE patients without previous cardiovascular disease were randomized to receive atorvastatin (40 mg/day) or placebo. Helical-CT scanning for coronary artery calcium measurement and cIMT and plaque detection were performed at baseline and after 2 years, but no significant difference was found between the groups in terms of progression of these variables. Similar negative results have also been obtained in SLE pediatric populations (156).

#### ACEIs and Angiotensin II Receptor Antagonists (ARBs)

The renin–angiotensin–aldosterone system (RAAS) has been implicated in atherogenesis (191). In a SLE mice model, an enhance vasoconstriction response to the RAAS which promotes vascular changes during the SLE course was noticed (192). Despite this possible contribution for atherosclerosis development, only few studies have focused on the potentially protective role of ACEIs and ARBs in SLE. A non-randomized prospective trial, comprising 144 patients with lupus nephritis treated with ACEIs/ARBs and 301 non-treated patients, showed no significant differences in the cumulative occurrence of CVE between the groups (160). One cross-sectional study comprising 51 SLE patients, mainly African-American, of whom only 12 were being treated with ACEIs, showed a strong association between total plaque area and 25(OH)-vitamin D insufficiency or ACEI non-use (161).

#### Aspirin

In healthy women, aspirin had no significant effect on the risk of myocardial infarction or death from cardiovascular causes, with the exception of women 65 years of age or older (relative risk 0.66, 95% CI 0.44–0.97, *p* = 0.04) (193). In SLE, aspirin did not alter the level of several atherosclerosis biomarkers (homocysteine, high-sensitivity CRP, soluble VCAM-1, P-selectin, and thrombomodulin) (158). Moreover, in the SLE cohort of the SOLVABLE study, baseline aspirin was even correlated with progression of coronary artery calcifications (194). Nevertheless, the authors hypothesized that patients under aspirin were already identified as those who may benefit from aspirin use. The role of aspirin in patients with APS positivity should not be forgotten, as in these patients, aspirin reduces the cardiovascular risk and thrombotic events (164).

#### Hydroxychloroquine

Hydroxychloroquine (HCQ) has well-known benefits for lipid (166) and glycemic control (166, 167) and even reduces the risk of thrombovascular events (169, 170). In an animal model of SLE, early treatment with HCQ prevented the development of endothelial dysfunction *via* reduction of reactive oxygen species (165). Furthermore, HCQ has been shown to reduce the carotid plaque burden and aortic stiffness in SLE patients (170). One recent paper from Fasano and colleagues supported the association of HCQ and aspirin in patients with Lupus for primary prevention of CVE (162). The authors performed an observational study and multivariate analysis that lead to the conclusion that both aspirin and HCQ reduced the risk of the first CVE (hazard ratio 0.24 and 0.027, respectively), and they found a time-dependent effect of HCQ, as HCQ protective effect was only significant after 5 years of treatment. The same author found in a similar designed study that the association of aspirin to HCQ had a synergistic thromboprotective effect (163).

#### Mycophenolate Mofetil (MMF)

In mice, MMF slows down the progression of atherosclerosis by inhibiting CD4+ T-cell activation and infiltration to the atherosclerotic lesion (173). One interesting clinical study enrolled 22 SLE patients who were undergoing carotid endarterectomy and randomized them in two groups (171). One group received 1,000 mg of MMF for 2 weeks prior to the surgery, while the other group received placebo. When compared to the placebo group, the carotid plaques of the MMF group had reduced number of activated T-cells and increased number of regulatory T-cells and had also a reduced pro-inflammatory and metalloproteinase genes expression. Contrary to this trial, no beneficial effect of MMF in the progression of cIMT or coronary calcification was noted in a 2-year longitudinal cohort study, despite the fact that only 25 patients of the study received the drug and at variable doses (172).

#### Azathioprine

Several data point out that azathioprine is linked to a higher risk of cardiovascular disease (40, 138, 174, 175), but as for corticosteroids, azathioprine use is also associated with higher disease activity which may cofound the results.

#### Cyclophosphamide and Cyclosporine

Studies on other less commonly used immunosuppressive drugs are scarce. The prevalence of abnormal aortic IMT and plaques was found to be negatively correlated with cyclophosphamide therapy (123). Similarly, current use of cyclosporine A was found to be protective against increased cIMT (148).

#### Monoclonal Antibodies

CD20-specific monoclonal antibodies administered to apoE−/− and LDLr−/− mice were shown to significantly decrease atherosclerosis, probably through reduction of the IgG type anti-oxLDL antibodies and through reduction in the accumulation of B-cells, macrophage, and T-lymphocytes in atherosclerotic plaques (180).

Mice treated with antibodies against the B-cell activating factor receptor (BAFFR) or that lacked the BAFFR exhibited reduced atherosclerosis (176). This was postulated to be related with the consequent depletion of the atherogenic B2 cells subtype and the preservation of B1 cells subtype. This last subtype is considered atheroprotective, as it produces IgM antibodies against oxLDL and apoptotic cells (177, 178). BAFF inhibition also prevents APS in lupus-prone mice, suggesting it could have a potential benefit in preventing thrombosis in patients with SLE (179).

#### Vitamin D

Vitamin D regulates several important immune functions and its deficiency has been linked to premature atherosclerosis (182–185). In apolipoprotein E knockout mice, calcitriol treatment changes the function or differentiation of dendritic cells and regulatory T-cells, decreases the production of pro-inflammatory chemokines, and reduces the quantity of inflammatory effector cells in atherosclerotic plaques (181). For patients with SLE, however, evidence supporting the supplementation with vitamin D to reduce atherosclerosis progression are lacking. In fact, several studies failed to prove a protective effect in atherosclerosis (96–98).

#### Lifestyle Modifications

Smoking cessation should be recommended for all SLE patients, because smoking is a strong predictor of cardiovascular diseases with an odds ratio of 3.731 (CI 1.39–10.0) (195).

To our knowledge, only one non-randomized small-scale study analyzed the effect of supervised physical exercise on the endothelial function and ergospirometric test variables in SLE. Improved FMD, exercise tolerance, and threshold velocity after training was noted in the group of physical exercise (196).

#### Future Perspectives

No strong conclusion can be drawn from the currently available literature concerning the effects in atherosclerosis of most of the currently used drugs in SLE. Despite the lack of recommendations, it is prudent for clinicians to treat modifiable risk factors whenever possible.

Understanding that atherogenesis is an inflammatory process by nature is important for the future development of drugs aimed at stopping or reversing plaque formation. A meta-analysis concluded that regression/progression of cIMT induced by several drugs did not predict changes in the occurrence of major CVE (197), therefore, addressing both the reduction of atherosclerosis surrogate markers and CVE will be crucial for treatment recommendations.

Celastrol is a potential future candidate for slowing down atherosclerosis in SLE. Although not used currently for the treatment of SLE, the effect of this drug was tested in active chromatin-induced SLE BALB/c mice, where it was shown to improve proteinuria, lower anti-nuclear, and anti-dsDNA antibodies, reduce renal histological changes, and increase survival rate (198). In addition, celastrol inhibits several important atherosclerosis pathways such as the production of LDL, expression of VEGF, formation of lectin-like oxidized LDL receptor-1, and reactive oxygen species (199, 200). Plaque ratio reduction was achieved with this drug in a rabbit experimental model (199).

The 4-F peptide, an apoA-I fragment, was shown to convert HDL from pro-inflammatory to anti-inflammatory, prevent the inflammation induced by oxidized lipids and to dramatically reduce atherosclerosis in mice, suggesting that it could also be used in the future for the prevention of atherosclerosis (201, 202).

We have showed before that IL-1 is involved in atherosclerosis development, thus targeting the IL-1 pathway could be a potential therapeutic approach against atherosclerosis in patients with SLE. The Canakinumab Anti-Inflammatory Thrombosis Outcomes Study trial provided evidence that targeting IL-1β with canakinumab (150 mg every 3 months) was associated with significant lower incidence of nonfatal myocardial infarction, nonfatal stroke, or cardiovascular death (hazard ratio versus placebo, 0.85, *p* = 0.02) (203). The higher incidence of fatal infections noted in the canakinumab group, the lack of an established role in the treatment of SLE and their expensive cost will, however, limit the use of IL-1 blockers for preventing atherosclerosis in SLE.

If promising results are obtained in these trials, subsequent studies in SLE may be warranted.

## Conclusion

We presented the current state of the research in premature atherosclerosis in SLE.

In SLE, cardiovascular diseases are the main cause of death, with risk ratios for several CVE (myocardial infarction, stroke, and peripheral artery disease) overcoming that of the general population. In our review, systemic inflammation was shown to be the cornerstone of the pathophysiological process of atherogenesis in lupus. New contributions for atherosclerosis development have been highlighted in recent years, namely, the role of EPCs, OxLDL and OxLDL autoantibodies, NETosis, and piHDL. Additionally, a raising number of molecular biomarkers have been proposed, but a direct comparison of these markers in terms of atherosclerotic events prediction risk has not been done so far. Biomarkers and risk factors integrated in panels are a promising strategy for enhancing CVD risk prediction, but they need to be validated in other cohorts.

We have also shown several conflicting results regarding lupus-related risk factors, namely, disease activity scores and the protective/harmful effect of several drugs, with most of the existing studies being observational. Different variable definitions may contribute to these differences. For example, many studies only evaluated the presence/absence of a drug as a CVD risk factor, others considered the highest dose or the cumulative dose; another example is the significant heterogeneity in IMT measurements among studies. In the future, standardization of variables definitions and of the imaging methods for evaluation of atherosclerosis should be sought according to their greatest clinical utility.

There is still a great need for controlled prospective studies for evaluating the efficacy of drugs in reducing not only atherosclerosis surrogates, but also CVE. So far, conventional treatment strategies for CVD, such as statins and ACEIs/ARBs, have not proved to be effective in preventing to reduce atherosclerosis progression in SLE. On the other hand, some immunomodulating agents, such as HCQ have demonstrated several favorable effects in CVE prevention, suggesting that disease-related factors may be more important in lupus atherogenesis than conventional risk factors and that future treatment strategies shall address systemic inflammation.

In conclusion, SLE and atherosclerosis have a multifactorial nature and an intricate relationship that has been considerably studied in the literature. Nevertheless, the current knowledge remains insufficient, as from a clinical point of view, it has not yet translated into effective approaches for prevention of both atherosclerosis progression and CVE and related mortality.

## Author Contributions

All authors wrote the main manuscript text and approved the final version.

## Conflict of Interest Statement

The authors declare that the research was conducted in the absence of any commercial or financial relationships that could be construed as a potential conflict of interest.

## Abbreviations

ACEI, angiotensin-converting enzyme inhibitor; Apo-AI, apolipoprotein A1; aPL, antiphospholipid antibodies; APS, antiphospholipid syndrome; ARB, angiotensin II receptor antagonist; BAFFR, B-cell activating factor receptor; cIMT, carotid intima-media thickness; CRP, C-reactive protein; CVE, cardiovascular events; DM, diabetes mellitus; dsDNA, double-stranded DNA; EPC, endothelial progenitor cell; FABP4, fatty-acid-binding protein 4; FMD, flow-mediated dilation; HCQ, hydroxychloroquine; ICAM-1, intercellular adhesion molecule 1; IFN, interferon; IL, interleukin; IMT, intima-media thickness; LDL, low-density lipoprotein; MCP-1, monocyte chemotactic protein-1; MMF, mycophenolate mofetil; MRI, magnetic resonance imaging; oxLDL, oxidized low-density lipoprotein; PAOD, peripheral arterial occlusive disease; PCI, percutaneous coronary intervention; piHDL, pro-inflammatory high-density lipoprotein; PON-1, paraxonase 1; PTX3, pentraxin-3; PWV, pulse wave velocity; RAAS, renin–angiotensin–aldosterone system; sCD40L, soluble CD40 ligand; SLE, systemic lupus erythematosus; SPECT, single photon emission computed tomography dual isotope; TGF, transforming growth factor; TNF-α, tumor necrosis factor α; TWEAK, tumor necrosis factor-like weak inducer of apoptosis; VCAM-1, vascular cell adhesion molecule-1; VEGF, vascular endothelial growth factor; vWf, von Willebrand factor.
